# The complete chloroplast genome sequence of *Vitis pseudoreticulata*

**DOI:** 10.1080/23802359.2019.1676669

**Published:** 2019-10-18

**Authors:** Chao Ma, Peining Fu, Lei Wang, Liping Zhao, Wenping Xu, Caixi Zhang, Shiping Wang, Jiang Lu, Shiren Song

**Affiliations:** a Department of Plant Science, School of Agriculture and Biology, Shanghai Jiao Tong University, Shanghai, PR China;; b Center for Viticulture and Enology, School of Agriculture and Biology, Shanghai Jiao Tong University, Shanghai, PR China;; c Institute of Agro-food Science and Technology/Key Laboratory of Agro-products Processing Technology of Shandong, Shandong Academy of Agricultural Sciences, Jinan, PR China

**Keywords:** *Vitis pseudoreticulata*, chloroplast genome, Illumina sequencing, phylogenetic analysis

## Abstract

*Vitis pseudoreticulata* is a wild *Vitis* species distributed in Southern China and wildly used to crossbreeding based on its resistance to moisture. In this study, the complete chloroplast genome of *V. pseudoreticulata* was assembled for the first time. The chloroplast genome was 161,065 bp in length, including two single-copy regions (19,083 and 89,276), which separated by a pair of inverted repeat regions. In totally, 132 genes were predicted, including 87 CDSs, 8 rRNA genes and 37 tRNA genes. The phylogenetic tree analysis showed that *V. pseudoreticulata* was the closest related to *Vitis amurensis*.

*Vitis pseudoreticulata* is wild germplasm resources with a high resistance to moisture, which mainly distributed in the temperate regions of China, such as Henan, Jiangsu, Jiangxi, and Guangxi provinces (Wan et al. 2008). *Vitis pseudoreticulata* is also characterized by high resistance to anthracnose and white rot (Li et al. 2008). In this study, the complete chloroplast genome sequence of *V. pseudoreticulata* was sequenced for the first time, and the genome sequence was updated to the NCBI GeneBank database (MN149911). It offers useful information for the resistance breeding of grapevine.

Genomic DNA was extracted from leaves of wild *V. pseudoreticulata* grown at the National Grape Germplasm Repository (113°70′E; 34°72′N), Zhengzhou (stored in Centre for Viticulture and Oenology, Shanghai Jiao Tong University, Accession number: *V. pseudoreticulata*), China. The Rapid Plant Genomic DNA Isolation Kit (Sangon Biotech, Shanghai, China) was used to extract gDNA. HiSeq Xten PE 150 sequencing platform (Illumina, San Diego, CA) was used to do DNA sequencing. A total of 3.81 Gb clean reads data was obtained. SOAPdenovo version 2.04 software was used to assemble the complete chloroplast genomic (Luo et al. 2012), and the *V. vinifira* chloroplast genome sequences (DQ424856) was used as a reference (Jansen et al. 2006). GeSeq were used to annotate gene (Tillich et al. 2017).

The *V. pseudoreticulata* chloroplast genome was 161,065 bp in length, including a 19,083 bp small single-copy region and 89,276 bp large single-copy region, which separated by two inverted repeat regions. Gene annotation showed that the chloroplast genome contained 132 single genes, which included 87 protein-coding (CDS), 8 rRNA, and 37 tRNA genes. Most of the genes are single copy in the chloroplast genome, while 4 PCGs, 13 tRNAs, and four rRNA have two copies. And two PCGs (rps12, ycf12) have three copies, three tRNAs have four copies (trnM-CAU, trnN-GUU, and trnY-GUA).

To clarity, the phylogenetic position of *V. pseudoreticulata* within the Vitaceae, a neighbour-joining (NJ) phylogenetic tree was constructed by using 15 *Vitis* species through the MEGA X (Kumar et al. 2018). Fifteen *Vitis* species are clustered into two orders, that are Euvitis planch and Muscadinia planch (Figure 1), which consistent with the previous report (Xie et al. 2017). The *V. pseudoreticulata* in the phylogenetic tree was phylogenetically closer to *V. amurensis*. And it closer to species from Asia and Europe rather than from Americas within the family Vitaceae.

**Figure 1.**
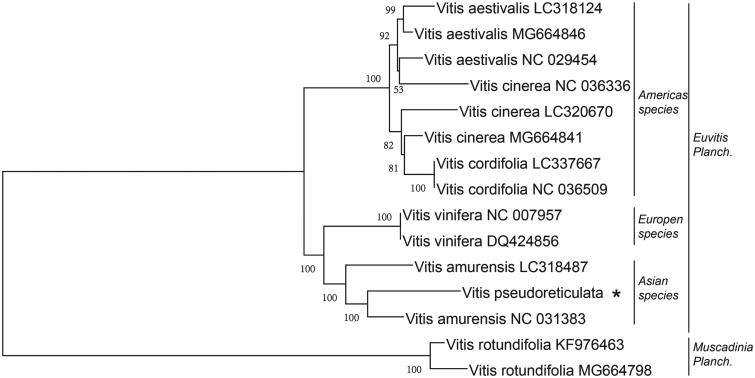
The phylogenetic tree of 15 species within the *Vitaceae* was constructed base on the neighbour-joining (NJ) analysis of chloroplast genomes (LSC, IRA, and SSC regions). The bootstrap values were based on 500 repetitions, and were shown next to the branches. The star indicates the species sequenced in this study, for easy reading by the reader.
